# Peroxiredoxin 6 Alone or in Combination with Fingolimod Ameliorates EAE

**DOI:** 10.2174/011570159X372166250619064636

**Published:** 2025-07-07

**Authors:** Sergey M. Lunin, Elena G. Novoselova, Olga V. Glushkova, Svetlana B. Parfenyuk, Anna A. Kuzekova, Tatyana V. Novoselova, Mars G. Sharapov, Elvira K. Mubarakshina, Ruslan G. Goncharov, Maxim O. Khrenov

**Affiliations:** 1 Institute of Cell Biophysics RAS, Pushchino, Moscow Region, Russia

**Keywords:** Multiple sclerosis, EAE, blood-brain barrier, peroxiredoxin 6, fingolimod, autoimmune response

## Abstract

**Introduction:**

Multiple Sclerosis (MS) is characterized by the infiltration of leukocytes into the nervous tissue, and disruption of the Blood-Brain Barrier (BBB) is one of the main factors in the progression of MS and its model, Experimental Autoimmune Encephalomyelitis (EAE). Furthermore, some anti-lymphocytic drugs against MS may inherently produce BBB disruption as their side effect. This study hypothesized that drugs restoring the BBB may be useful for the treatment of MS and EAE, as well as for ameliorating the side effects of modern anti-lymphocytic drugs.

**Methods:**

EAE was induced in *SJL/J* mice. EAE progression was evaluated by a severity score and a plasma cytokine profile, while a BBB condition was evaluated by the Evans dye method, Tight Junction Proteins (TJPs) content, and leukocyte infiltration.

**Results:**

The mice with EAE demonstrated neurological symptoms, a cytokine response, and BBB deterioration, which was associated with upregulation of the NADPH oxidases NOX1 and NOX4 in the brain. Administration of the anti-lymphocyte drug fingolimod to EAE mice caused lymphopenia, improved animal health, enhanced the BBB function during the administration period, and decreased the pro-inflammatory response, but it was accompanied by a “withdrawal effect,” defined as a sharp increase in the IL-17 and IFN-gamma to levels higher than those in untreated animals, lymphocyte hyperactivation, worsening symptoms, and increasing BBB permeability after discontinuation of fingolimod. Administration of peroxiredoxin 6 (Prdx6) to EAE mice also improved BBB, decreased lymphocyte infiltration and NADPH oxidase expression, and ameliorated symptoms. Preliminary administration of Prdx6 before the fingolimod treatment eliminated the “withdrawal effect” of fingolimod and led to full recovery of the EAE mice. This Prdx6 effect was associated with the activation of anti-proliferative and pro-apoptotic signaling cascades in lymphocytes.

**Discussion and Conclusion:**

Both fingolimod and Prdx6 produced beneficial effects, while Prdx6 may be useful for ameliorating the side effects of anti-lymphocytic drugs. Accounting for literature data that discontinuation of MS treatment is very likely to lead to a severe MS rebound, a drug that prevents the rebound should be useful.

## 1 INTRODUCTION

One of the most severe and incapacitating diseases of the modern world is Multiple Sclerosis (MS), which is an autoimmune disorder that primarily affects the white matter of the brain and spinal cord. Its progression results in many symptoms related to damaged neural tissues, particularly axonal myelin membranes. Myelin lesions disturb neuronal impulse conduction, leading to many neurological consequences that ultimately may result in paralysis and death. The main cause of such damage is T-lymphocytes, penetrating through the impaired Blood-Brain Barrier (BBB) and inducing local inflammatory reactions in neural tissue. This local inflammation impedes restoration of the BBB and may lead to additional invasion of auto-aggressive lymphocytes into the CNS. The BBB is formed by brain capillary endothelial cells, astrocytes, pericytes, and microglial cells [1, 2]. The endothelial cells are held together by two types of junctions: adherent junctions and tight junctions. The adherent junctions perform the function of maintaining cell-to-cell contacts and are also attached to the cytoskeleton. Tight junctions seal the spaces between cells, determine cell membrane polarity, and limit the permeability of the BBB. TJs form a sort of zipper that closes inter-endothelial spaces [3, 4]. They consist of membrane-tight junction proteins (TJPs), such as occludins and claudins, and membrane-associated proteins, such as Zonula Occludens (ZO-1) [5]. The role of these proteins is of paramount importance for BBB formation [6-10], and, indeed, alterations in their expression can be pathogenic [11, 12].

A severe systemic inflammation may induce the initial BBB breakage. The inflammatory reaction in response to bacteria, for example, may lead to massive ROS production, while the large quantities of ROS were demonstrated to induce pathological changes in vascular endothelial cells of CNS, which form the BBB, weakening tight junctions and affecting their morphology. These changes may allow lymphocytes to enter CNS tissue and produce an auto-aggressive reaction to myelin. Indeed, the Experimental Autoimmune Encephalomyelitis (EAE), which models MS, is induced by this scheme. Particularly, a pertussis toxin, which is known to compromise BBB integrity [13], is commonly used for EAE induction. In multiple sclerosis, BBB disruption is evident in lesions [14]. Therefore, critical factors for the induction and progression of multiple sclerosis are the presence of autoimmune aggressive lymphocyte populations and the lack of integrity of the BBB. Moreover, the brain astrocytes are able to interact with self-reactive lymphocytes and adapt themselves by assuming an inflammatory phenotype [15, 16]. Therefore, we hypothesized that drugs, improving the BBB condition may be beneficial in MS.

Currently, the cutting-edge drugs used to treat multiple sclerosis are anti-lymphocyte drugs, such as sphingosine-1-phosphate (S1P) receptor modulators (*e.g*., fingolimod), which prevent the exit of lymphocytes from peripheral lymphoid organs [17]. Initially, sphingosine-1-phosphate was considered an inert product of sphingosine catabolism. However, with the discovery of ubiquitous S1P receptors, the concept of S1P as an extracellular primary messenger with autocrine and paracrine actions has emerged. The binding of S1P to corresponding receptors leads to a variety of cellular responses, including proliferation, survival, invasion, adhesion, and migration; thus, S1P is very important for the normal functioning of blood vessels and nervous, immune, and other systems [18-20].

A nonselective S1P1 receptor modulator called fingolimod has undergone clinical trials and was approved in the USA and Russia in 2010/11 for the treatment of the relapsing-remitting form of MS. Although fingolimod is actually an agonist of S1P1 receptors, it is also a functional antagonist since activation of the receptor leads to its internalization and cleavage [21-23]. Since the directed migration of lymphocytes depends on the expression of S1P1 receptors on their surface, the degradation of S1P1 leads to the retention and accumulation of lymphocytes in lymphoid organs and a decrease in their number in the blood [17], which is the basis for the therapeutic effect of this drug. However, S1P1 has a pleiotropic effect, and the integrity of the BBB depends on S1P signaling; thus, S1P1 blockers may impair vascular barrier function [24]. Certainly, fingolimod can cause BBB breakage [25], which is clearly unfavorable under conditions of lymphocyte infiltration into the central nervous system. Although fingolimod is approved for the treatment of MS, it does not provide a complete cure, and the results have been ambiguous in large cohorts of patients, as described in a review of La Mantia with co-authors [26]. The main finding of this review was that fingolimod 0.5 mg daily as monotherapy increased the probability of relapse-free survival at 24 months compared with placebo. However, no effect was found in preventing progressive disability. A greater probability of treatment discontinuation was revealed due to adverse events at short follow-up (6 months) with fingolimod than with immunomodulatory drugs; compared with interferon beta, no significant differences were found 12 months after treatment. The duration of all studies was 24 months or less, so the effectiveness of fingolimod (and its general safety) in the longer term (more than 24 months) remains uncertain. However, this perspective is extremely important for a lifelong disease that requires ongoing treatment, such as MS. Currently, extensive efforts are underway to test selective modulators of S1P receptor subtypes [27] as well as to identify compounds that target S1P receptors in conjunction with other receptor systems [28]. However, it should be noted that the advantageous effect of S1P on lymphocytes and the undesirable effect of S1P on the BBB can be achieved through the same S1P1 receptor. This feature may be a factor limiting the effectiveness of selective modulators.

As mentioned earlier, ROS plays an important role in the onset and progression of MS and EAE as its model. Monocytes and other cell types produce ROS and induce changes in the vascular endothelium, which allows lymphocytes to enter the neural tissue [29-31]. Lymphocytes induce local inflammatory reactions in the CNS, preventing the restoration of the BBB and inducing the breakdown of myelin sheaths by macrophages [32-34]. In addition, upon contact with myelin proteins in a pro-inflammatory environment, auto-aggressive populations of lymphocytes may be raised and supported, which contributes to the long-term maintenance of the inflammatory process.

Antioxidants have repeatedly been shown to protect against inflammation by lowering ROS levels and may exert a protective effect in various models of inflammation [35-37]. However, the results of clinical studies of antioxidants in relation to neurological diseases were ambiguous [38]. Recent findings showed that vascular endothelial cells proximate to myelin lesions in MS or EAE express peroxiredoxin 6 (Prdx6), probably as an adaptation [39]. This protein, from the family of peroxiredoxins, has both peroxidase and phospholipase activity and plays an important role in protection against oxidative stress, the restoration of membranes after oxidation, the activation of some NADPH oxidases, and signaling cascades associated with oxidative stress [40, 41]. Our recent data demonstrated that Prdx6 may protect pancreatic β-cells in a model of diabetes mellitus *in vitro* [42], as well as in alloxan-induced type 1 diabetes in mice [43]. Expression of Prdx6 in brain tissues was shown to be important for brain functioning, for example, for synaptic plasticity [44], and related to CNS disorders [45]. Prdx6, alone among all peroxiredoxins, is expressed in mouse astrocytes, and it was shown to have increased expression in the spinal cord of mice with induced EAE. Additionally, a significant reduction in EAE symptoms and demyelination severity was showed in mice with over-expression of Prdx6 compared to wild type, whereas increased expression of this peroxiredoxin in astrocytes in mice with EAE, as well as in humans with multiple sclerosis resulted in down-regulation of the metalloproteinase MMP9 (greatly involved in BBB disruption). Along with that, chemokine levels and ROS levels were decreased, resulting in improved BBB condition and reduced lymphocyte infiltration [39]. Prdx1, another peroxiredoxin, improved the integrity of the endothelial cell layer *in vitro*, and its expression was upregulated in rats with EAE [31]. Prdx5 and Prdx6 levels were elevated in patients with MS and neuromyelitis optica, correlating with clinical parameters. Moreover, the upregulation of Prdx5 and Prdx6 correlated with BBB-mediated disorders [46]. We hypothesized that the introduction of Prdx6 protein may decrease ROS levels and trigger the reparation of the BBB. Restoring the BBB should prevent access of lymphocytes to the neural tissue and break the vicious circle of autoimmune inflammation.

To test this hypothesis, we recently assessed the effect of exogenous Prdx6 on the BBB and showed that Prdx6 could indeed have a beneficial effect on the BBB, reducing its permeability under conditions of induced EAE, reducing the infiltration of lymphocytes into the spinal cord and improving the health status of animals [47]. The present work is a continuation of the above study, and we plan to test further the effect of Prdx6 on the state of the BBB and the progression of the multiple sclerosis model and, additionally, to evaluate the possibility of neutralizing the side effects of fingolimod, a modulator of S1P1 receptors, which may limit the effectiveness of its use. These findings suggest that coadministration of Prdx6 in combination with fingolimod may improve the results of anti-lymphocyte therapy.

## 2 MATERIALS AND METHODS

### 2.1 Experimental Animals

Female *SJL/J* mice (SPF Breeding Facility for Laboratory Animals, Pushchino, Russia) with the age of 3 months and weight in the range of 20-24 grams were kept 4 per cage under standard laboratory conditions (temperature 20-22°C, 12 hours light/12 hours dark condition). Standard mouse food pellets and water were provided ad lib. The experimental procedures were performed in accordance with the Guidelines for Ethical Conduct in the Care and Use of Animals and were approved by the Ethical Committee of the Institute of Cell Biophysics, Pushchino, Russia (approval #57, 12/30/2011).

### 2.2 Induction of EAE and Drug Administration

A total of 150 μg/mice of Proteolipid Protein (PLP) peptide, amino-acids 139-151 (GenScript, USA) was dissolved in sterile saline and then emulsified in an equal volume of fortified complete Freund's adjuvant containing 4 mg/mL *Mycobacterium tuberculosis,* strain H37Ra (Chondrex, USA). The emulsion was injected (3 × 30 μL) subcutaneously into 3 sites along the vertebral column. Immediately after PLP injection (Day 0) and 48 hours thereafter (Day 2), 400 ng/mice of pertussis toxin (US Biological, USA) was injected intraperitoneally. Mice were observed individually until the first signs of EAE developed and then randomized into the experimental groups using a simple randomization protocol. The mice without visible EAE signs were excluded from the experiment. To quantitatively measure EAE symptoms, the following scale was used: score 0, no symptoms; score 1, limp tail; score 2, wobbly gait; score 3, hind limb weakness; score 4, hind limb paralysis; and score 5, tetraparalysis/ death. There were six groups with 20 mice/ group: control, Prdx6, EAE, EAE+Prdx6, EAE + fingolimod, and EAE+ Prdx6+fingolimod. To minimize potential influences of the order of treatments/measurements, regular assessments and drug treatments were performed using a random order of cages and a random order of animals in each cage. The animals were sacrificed by decapitation in subgroups of 10 animals on Days 16 and 28. Among them, 3 animals per subgroup were taken for BBB permeability analysis by the Evans blue dye method. Sample sizes were selected so that each group included at least three, although usually 4 animals are included for each result data comparison (n=3-4 biological replicates). The sample size was calculated by a resource equation method [48]. E value was 36 (>20).

Considering that fingolimod has a demonstrated effect on multiple sclerosis, the application of fingolimod (MiraxBioPharma, Russia) (intraperitoneally, 0.2 mg/kg per injection) began at the onset of the first symptoms in most mice (to avoid possible interference with EAE development), on 12^th^ day after induction, and was injected every other day for one week (total of 3 injections).

Prdx6 was synthesized in our laboratory using genes encoding wild-type mouse *Prdx6*, cloned and expressed in *E. coli* BL21 (DE3) as described earlier [49]. The protein was purified *via* metal-affinity chromatography as describer earlier [50].

Prdx6 was administered (6 mg/kg) i.p. on Days 2, 7, and 10. The dose and route of administration were adapted from our previous studies [49] and preliminary experiments as the most effective with the least side effects.

### 2.3 BBB Condition

The permeability of the BBB was detected *via* quantitative measurement of Evans Blue dye (EB) accumulation in nervous tissue, as described previously [51]. Briefly, mice on Days 16 and 28 (n=3 per subgroup) were sedated with i.p. injection of ketamine hydrochloride (80 mg/kg) and xylazine hydrochloride (10 mg/kg). Then, the mice were injected with 3 ml/kg Evans blue dye (EB, 2%) through the tail vein. After 1 hour, the mice were perfused with saline through the right atrium to remove vascular EB dye until the fluid flowing out of the right atrium was clear. Next, brains were removed, weighed, and frozen at -80°С until analysis. After thawing at 37°С, the brains were homogenized in 500 μL of 50% trichloroacetic acid. The homogenate was centrifuged for 20 min at 10,000 × g, and the supernatant was diluted 4-fold with ethanol. The absorbance of the supernatant was measured at 620 nm. EB concentration was calculated (in micrograms per milligram of brain tissue) using a standard curve of EB in ethanol. Accumulation of EB in the brain tissue was used as a measure of BBB breakage.

### 2.4 Blood Tests

Given that fingolimod is an anti-lymphocyte drug, all animals were monitored for lymphopenia *via* blood tests performed with a hematology analyzer BC-2800Vet (Mindray, China) to control the effect of fingolimod. Blood samples for the analysis were obtained at the time of sacrifice (Days 16 and 28). Lymphopenia was used as a confirmation of the anti-lymphocytic effect of fingolimod.

### 2.5 Experimental Samples

The animals from each experimental group were sacrificed in 2 subgroups (time points) on days 16 and 28, and the following experimental samples were collected. For Western blotting or immunohistochemistry, the brains and spinal cords were collected following decapitation and frozen at -80°С until analysis. For plasma cytokine measurements by ELISA (Enzyme-Linked Immunosorbent Assay), blood was collected during decapitation. Samples were kept for 3-5 hours at 4°С and centrifuged at 200 ×g. Plasma samples were collected by pipetting and analyzed immediately. For Western blotting analysis of signal proteins, spleen cells were isolated from the spleen in the RPMI 1640 medium (Sigma, USA). Erythrocytes were lysed in Tris-buffered ammonium chloride (0.01 M Tris-HCl with 0.15 M NaCl and 0.83% NH4Cl at a ratio of 9:1). After washing, the cells were counted, diluted to a concentration of 1 × 10^8^ cells/mL in PBS and stored at −80 °C until analysis.

### 2.6 Histology and Immunohistochemistry

The mice were sacrificed on days 16 and 28 (n=3 per group) to evaluate the occludin-1 level and lymphocyte infiltration in the spinal cord. Spinal cords were harvested and fixed in 4% formaldehyde for 24 hours; 4-μm-thick transverse sections were prepared and stained with Hematoxylin-Eosin (HE) for inflammatory cell infiltration or with an anti-occludin-1 antibody conjugated to horseradish peroxidase (1:500, Affinity Bioscience, China). The antibody-bound sections were visualized with 3,3'-Diaminobenzidine (DAB) (Boster, China). The images of the stained sections were examined with an Olympus PX53 microscope with a DP72 digital camera attached. Images were evaluated using ImageJ software (National Institutes of Health, USA). Provided that occludin-1 accumulates mainly in the gray matter area but was almost absent in the white matter area, for anti-occludin-1-stained sections, the mean pixel intensities in the gray matter area (manual delineation) were normalized to the mean pixel intensities in the white matter area (manual delineation). For each animal, the values were counted and averaged in three sections (technical repeats). For HE-stained sections, the cells in regions of white matter were counted and averaged in three Fields Of View (FOVs). Histological analyses were performed IN A blinded manner with respect to treatment groups.

### 2.7 Plasma Cytokine Measurements

Plasma cytokine concentrations were determined using ELISA kits for mouse TNF-α, IL-6, IL-17, and IFN-γ (Cloud-Clone Corp., China). To visualize binding, 100 µL of ABTS green dye (Sigma) dissolved in 0.05 M citrate buffer (pH 5.0) with 0.01% hydrogen peroxide was added to the samples, and the optical density was measured at 405 nm using a microplate spectrophotometer (Multiskan EX; Thermo Electron Corporation, USA). Analyses were performed in a blinded manner with respect to treatment groups.

### 2.8 Western Blot Analysis of Signal Proteins and TJPs

The analysis of signaling proteins was performed using samples of spleen cells, while the analysis of tight-junction proteins was performed using whole-brain homogenates. For spleen cell analysis, 1 × 10^8^ splenic cells were lysed with Triton^®^ X-100 lysis buffer containing 50 mM Tris-HCl (pH 7.4), 150 mM NaCl, 1% Triton X-100, and 5 mM EDTA (Alfa Aesar, UK) and the cocktail of inhibitors of proteases (#G2006 ServiceBio, China), and phosphatases (#G2007 ServiceBio, China). For ЕОЗ analysis, brain samples were thawed and homogenized in RIPA buffer (HiMedia Laboratories, India) with the addition of the same cocktails of inhibitors. A Nanodrop spectrophotometer was used to measure the total protein content in each sample. The proteins were subsequently precipitated with acetone, solubilized in SDS buffer, boiled for 5 minutes, and stored at −80°C. The samples were applied at 10 μg of total protein per lane and resolved *via* electrophoresis on a 10% polyacrylamide gel. Afterward, proteins were transferred from the gel to a nitrocellulose membrane (Amersham/GE Healthcare, UK) in a Trans-Blot chamber (Bio-Rad, USA). The membrane was then blocked for 1 hour with Tris-buffered saline (TBS, pH 7.4) containing 5% of dry milk. Following blocking, the membrane was exposed for two hours to an antibody against one of the following proteins: phospho-NF-κB (rabbit anti-mouse phospho-NF-κB p65 (Ser536) antibody; Affinity Bioscience, China), total NF-κB (rabbit anti-mouse NF-κB p65 antibody; Affinity Bioscience, China), phospho-p53 (rabbit anti-mouse phospho-p53 (Ser46) antibody; Cell Signaling Technology), p53 (rabbit anti-mouse p53 (1C12) antibody; Cell Signaling Technology), cleaved caspase-3 (rabbit anti-mouse caspase-3 (8G10) antibody; Cell Signaling Technology), occludin-1 (rabbit anti-mouse occludin-1; Affinity Bioscience, China), ZO-1 (rabbit anti-mouse ZO-1; Affinity Bioscience, China). After washing, the membrane was incubated for 1 hour with a biotinylated goat anti-rabbit antibody (Biotin-SP AffiniPure goat anti-rabbit IgG; Jackson ImmunoResearch, USA) and then exposed to 0.1 µg/mL peroxidase-conjugated streptavidin (Jackson ImmunoResearch) for 1 hour. The antibody to mouse Glyceraldehyde-3-Phosphate Dehydrogenase (GAPDH) (rabbit anti-mouse GAPDH; Cloud-Clone Corp, China) was used to account for variations in protein loading. Clarity Western substrate chemiluminescence reagents (Bio-Rad/USA) and Hyperfilm ECL (Amersham/GE Healthcare) were then used to develop the blots. The developed films were observed with a Vilber LourmatTFX-35WL (France) transilluminator. The protein bands were subsequently quantitatively evaluated using QAPA software v. 3.7 (Russia). Analyses were performed blind to treatment groups. ph-NFkB/NFkB ratio was used as a measure of splenocyte activation, ph-p53/p53 ratio, and cleaved caspase-3 were used as a measure of activation of anti-proliferative/pro-apoptotic pathways, ZO-1 and occludin-1 were used as a measure of TJPs washout and BBB breakage.

### 2.9 Gene Expression Analysis

Isolation of total RNA from brain tissue, synthesis of cDNA, and real-time PCR were performed as described previously [52]. The expression of the genes was normalized to a housekeeping gene, ribosomal protein lateral stalk subunit P2 (*Rplp2*). Changes in gene expression were normalized to the control values of intact mice. ΔCt values were calculated using the formula ΔCt = Ct (gene of interest) – Ct (Rplp2); ΔΔCt was calculated as ΔCt (experiment) – ΔCt (control). To calculate differences in gene expression, the 2^ - ΔΔCt method was used [52]. Analyses were performed blind to treatment groups.

### 2.10 Statistics

Statistical analyses were performed using Statistica 6.0 software (StatSoft, USA). The Shapiro-Wilk test was used to check the normality of the distribution. The W value was not significant in any case (*p* > 0.05). Two-way (for the changes of severity score and body weight) or one-way analysis of variance (for all other measurements) with post hoc Tukey tests were used to determine the significance of differences among the groups. The *p* values ≤0.05 were considered significant. All values are expressed as the mean ± standard error of the mean.

## 3 RESULTS

### 3.1 Induction and Progression of EAE

**Fig. (1):**
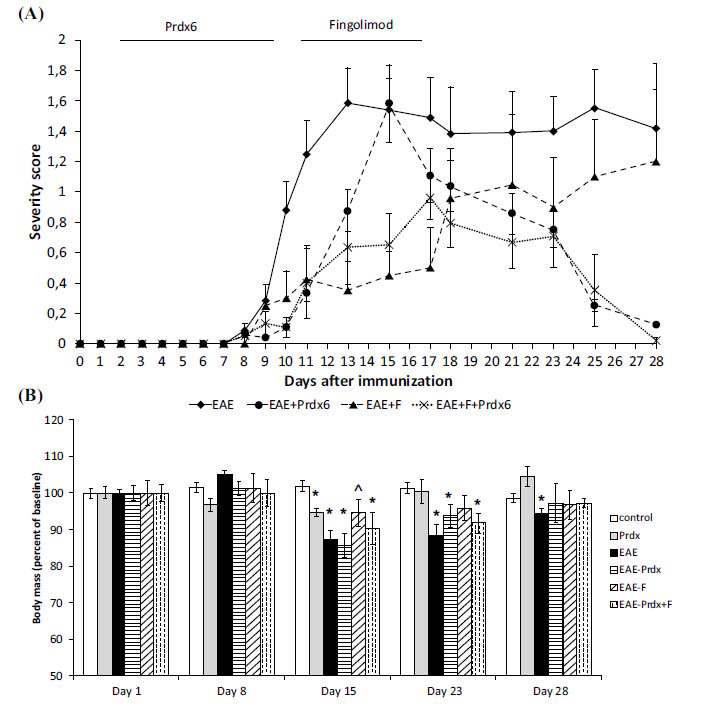
Effects of Prdx6 and/or fingolimod on EAE progression. (**A**). EAE symptom severity was assessed using the following scale: 0, normal; 1, limp tail; 2, wobbly gait; 3, hind limb weakness; 4, hind limb paralysis; 5, tetraparalysis/death. Experimental groups: EAE, EAE+Prdx6, EAE + fingolimod and EAE+Prdx6+fingolimod. The time bars represent periods of Prdx6 and fingolimod administration, where applicable. (**B**). Percent body weight changes. Experimental groups: healthy mice, Prdx6, EAE, EAE+peroxiredoxin-6, EAE + fingolimod, and EAE+peroxiredoxin-6+fingolimod. The values were calculated individually for each mouse at specified time points after immunization (day 0) relative to the individual baseline weights (day -2). The baseline mean weights of the mice in all the groups were in the range of 21.4 ± 0.5 g. In both Panels A and B, the data are presented as the means ± SEMs for 20 mice (days 0-16) or 10 mice (days 16-28) per group. *Significant difference from the control group, *p* < 0.05. #Significant difference from the EAE group, *p* < 0.05.

A relapsing-remitting form of Experimental Autoimmune Encephalomyelitis (EAE), a model of multiple sclerosis, was induced according to a well-recognized regimen previously proposed for this model. EAE led to impaired myotonus, motor dysfunctions, or paralysis. In the present study, EAE emerged in 58 (97%) of the 60 mice. The first signs of EAE appeared on day 8, which was consistent with the existing literature. The most frequent signs of EAE in our setting were loss of tail muscle tone and hind limb paralysis, although more severe signs were also observed (Fig. **1A**). Additionally, the mice with EAE demonstrated weight loss, especially at the first stages of the disease, with slow and incomplete restoration (Fig. **1B**).

Fingolimod monotherapy led to an improvement in the general health status of the animals. Symptoms that developed were less severe than those in the untreated group, but after discontinuation of fingolimod (after day 17), symptoms began to worsen, and at the end of the observation period (day 28), they approached those of the untreated group. Symptoms of the animals in EAE+Prdx6+fingolimod weakened, similar to those in the EAE+fingolimod group, but after the discontinuation of fingolimod, their symptoms not only did not worsen but even improved until almost complete recovery (Fig. **1A**).

Thus, although its administration began and ended before the administration of fingolimod and did not interfere with the onset of disease symptoms itself, Prdx6 prevented the return of symptoms when fingolimod was discontinued. With respect to body weight, all EAE groups treated with fingolimod, peroxiredoxin 6, or peroxiredoxin 6 in combination with fingolimod regained weight to levels no different from those of the controls more quickly than did the untreated group. Thus, the combination of peroxiredoxin with fingolimod was clearly beneficial both on its own and in comparison with fingolimod monotherapy.

### 3.2 Leukocyte Status

**Fig. (2):**
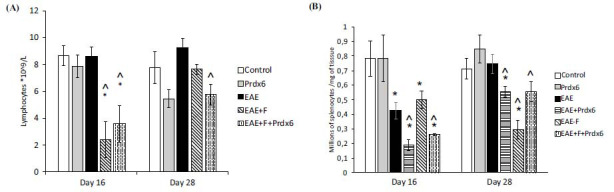
Effects of Prdx6 and/or fingolimod on leukocyte status. (**A**). Blood leukocyte count. Experimental groups: healthy mice, Prdx6, EAE, EAE + fingolimod, and EAE+Prdx6+fingolimod. (**B**). Spleen cell count. Experimental groups: healthy mice, Prdx6, EAE, EAE+peroxiredoxin-6, EAE + fingolimod, and EAE+peroxiredoxin-6+fingolimod. In both Panels (**A** and **B**), the data are presented as the means ± SEMs for 10 mice per group. Measurements were obtained at different times after immunization (day 0). *Significant difference from the control group, *p* < 0.05. ^Significant difference from the EAE group, *p* < 0.05.

Since fingolimod is an anti-lymphocyte drug that induces lymphopenia, the level of lymphocytes in the blood was monitored in the animals of all the experimental groups using a hemoanalyzer. Blood samples for analysis were obtained on days 16 and 28 when the animals were sacrificed. At the same time points, the content of splenocytes in the spleen was also monitored for a general assessment of the state of the lymphocyte depot. The results (Fig. **2A**) revealed that neither EAE nor Prdx6 alone led to significant changes in blood lymphocyte counts (although Prdx6 injection produced some trend toward a decrease in lymphocyte counts at later time points, which did not reach significance). Induction of EAE caused a significant decrease in the number of cells in the splenic depot in the initial stages of the disease, with subsequent recovery to normal values (Fig. **2B**). A similar picture was observed when Prdx6 was administered to EAE-bearing animals (but with a lower cell count than that in the EAE group). The use of the anti-lymphocyte drug fingolimod caused a strong and significant decrease in the level of lymphocytes in the blood, and after stopping the administration of fingolimod (on day 17), the level of lymphocytes in the blood quickly returned to normal values. Additionally, the administration of fingolimod led to a gradual decrease in spleen cellularity throughout the observation period, which reflected the gradual depletion of the lymphocyte depot. Compared with fingolimod alone, the combination of fingolimod and Prdx6 significantly restored the spleen cell count in the late stage of EAE (Fig. **2B**).

### 3.3 Plasma Cytokine Levels

**Fig. (3):**
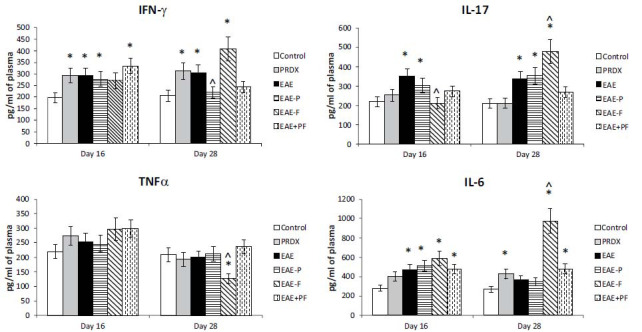
Blood cytokine profiles measured by ELISA. Experimental groups: healthy mice, Prdx6, EAE, EAE+Prdx6, EAE + fingolimod, and EAE+Prdx6+fingolimod. Measurements of cytokine content (IFN-γ, TNF-α, Il-6, and IL-17) were made *via* ELISA at different times after immunization (day 0). In all panels, the data are presented as the means ± SEMs for 10 mice per group. *Significant difference from the control group, *p* < 0.05. ^Significant difference from the EAE group, *p* < 0.05.

To assess the inflammation level in the mice with EAE, we measured cytokine concentrations in plasma samples *via* ELISA. The literature data suggests that T helpers 1 (Th1) and T helpers 17 (Th17) may play important roles in autoimmunity and are involved in multiple sclerosis and EAE [53]. We measured levels of cytokines produced primarily by Th1 and Th17 (interferon-γ and IL-17α, respectively), as well as IL-6 and TNF-α, cytokines produced by many immune cell populations, such as monocytes, macrophages, *etc*., which may be called more “universal” cytokines (Fig. **3**).

In blood plasma of mice with EAE, we observed a significantly elevated interferon-gamma (IFNγ) level, produced predominantly by Th1 cells, at early and later stages of disease progression, and the level of interleukin-17 (IL-17), produced predominantly by Th17 cells, which is consistent with earlier data on the role of Th1 and Th17 cells in multiple sclerosis and EAE as its model [47]. Interleukin 6 was elevated only in the earlier phase of EAE, while for tumor necrosis factor-alpha, no significant changes were demonstrated. Fingolimod monotherapy led to a decrease in the level of the proinflammatory cytokine IL-17 but not in the levels of IFN-gamma or IL-6 at the initial stage of the disease. Notably, a “withdrawal effect” of fingolimod was observed at the later stage, when fingolimod was no longer administered: we observed a sharp increase in the levels of IFN-γ, IL-17, and IL-6, which were higher than those observed in the untreated EAE-only group. Coadministration of fingolimod and Prdx6 led to a small but nonsignificant decrease in proinflammatory cytokines at the early stage of the disease, but at the later stage, the levels of all the cytokines studied were reduced to almost normal values. Thus, fingolimod therapy exerted an anti-inflammatory effect, accompanied by a proinflammatory “withdrawal effect.” In contrast, the administration of Prdx6 (which began and ended before the start of fingolimod administration) neutralized this withdrawal effect.

### 3.4 Signaling Pathways Activation in Spleen Immune Cells

**Fig. (4):**
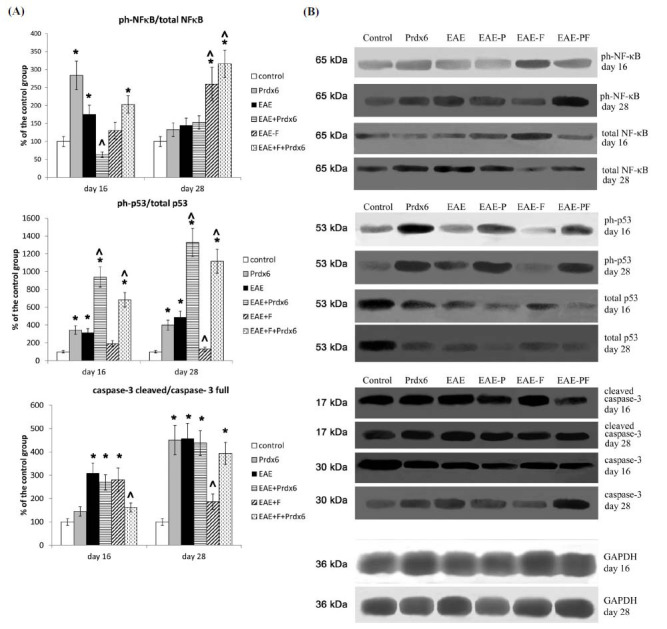
Signal cascade activation in spleen cells. Experimental groups: control (healthy mice), Prdx6, EAE, EAE+Prdx6, EAE + fingolimod, and EAE+Prdx6+fingolimod. Measurements were obtained *via* Western blotting at different times after immunization (day 0). (**A**) In the histograms, the densitometry data were normalized to the corresponding GAPDH loading control and the corresponding total protein content, then to the corresponding control group; the data are presented as the means ± SEMs for 3 mice per group. (**B**) The presented blot images are from a single representative animal per group. *Significant difference from the control group, *p* < 0.05. ^Significant difference from the EAE group, *p* < 0.05.

In addition, we measured the activation of signal pathways involved in the activation, proliferation, and apoptosis of immune cells to assess the status of immune cells during the EAE as well as the immunomodulatory effects of therapies (Fig. **4**).

The results revealed an EAE-induced increase in the phosphorylation of the p65 NF-kB protein, an important factor in lymphocyte activation. EAE also led to significant activation of cascades associated with the inhibition of proliferation and the induction of apoptosis (p53 and caspase-3, respectively). Activation of these cascades can lead to the arrest of proliferation and initiation of lymphocyte apoptosis, which may be an adaptogenic mechanism in the autoimmune response.

Compared with untreated EAE mice, the administration of fingolimod slightly reduced initial splenocyte activation, as assessed by NF-κB p65 phosphorylation. This phenomenon was expected, given the anti-lymphocyte effect of fingolimod. However, the withdrawal of fingolimod resulted in a dramatic increase in activation that exceeded the initial effect of EAE and was correlated with the “withdrawal effect” described above for the cytokine profiles. Additionally, in the EAE + fingolimod group, the activity of the antiproliferative (p53) and proapoptotic (caspase-3) cascades sharply decreased compared with that in the EAE group, *i.e*., the ability of the cells to proliferate and survive was restored.

Prdx6 alone (in healthy mice without EAE) significantly increased the activation of the NF-kB cascade but only at the early stage of the disease. By the late stage, the activating effect of Prdx6 disappeared. In EAE-bearing mice, the effect of Prdx6 was not significant. However, at all time points, Prdx6 had a significant stimulatory effect on the activation of the p53 cascade, which should contribute to the arrest of proliferation and, under some conditions, apoptosis, and this effect was additive with EAE. The activation of proapoptotic caspase-3 was approximately the same as that in untreated EAE mice.

Coadministration of fingolimod and Prdx6 to EAE mice increased the activation of proapoptotic (caspase-3) and antiproliferative (p53) cascades, which should be considered beneficial in conditions of autoimmune inflammation and the proliferation of autoaggressive clones of lymphocytes.

### 3.5 BBB Permeability

To identify other mechanisms underlying the observed effects of Prdx6 and fingolimod, we assessed the permeability of the Blood-Brain Barrier (BBB), which has previously been shown to be dysfunctional in both EAE and multiple sclerosis, *via* direct assessment of Evans dye permeation into brain tissue and alterations in Tight Junction Protein (TJP) content in the brain.

#### 3.5.1 Permeability Assay Using the Evans Blue Dye Method

**Fig. (5):**
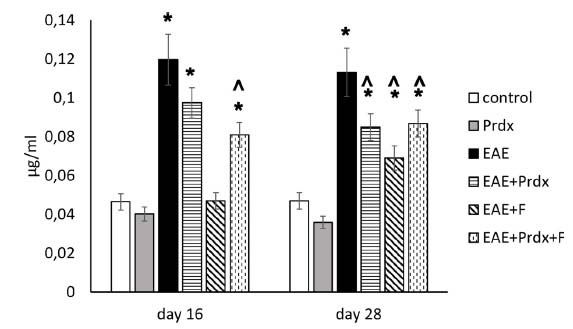
BBB permeability was measured by Evans Blue (EB) dye accumulation in brain tissue. Experimental groups: healthy mice, Prdx6, EAE, EAE+Prdx6, EAE + fingolimod, and EAE+Prdx6+fingolimod. The data are expressed in micrograms of EB dye per milliliter of brain tissue and are presented as the means ± SEMs for 3 mice per group. Measurements were obtained at different times after immunization (day 0). *Significant difference from the control group, *p* < 0.05. # Significant difference from the EAE group, *p* < 0.05.

To assess the permeability of the blood-brain barrier in the experimental mice, the accumulation of Evans blue dye in brain tissue was quantitatively measured following intravenous administration of the dye (Fig. **5**). Normally, an intact BBB should prevent Evans blue dye from entering into the brain tissue, and its accumulation in the brain indicates breakage of the BBB.

The data suggested that induction of EAE caused a dramatic disruption of BBB permeability, leading to a significant increase in EB dye accumulation in the brain. This increase was strong in both the early and later stages of EAE. Prdx6 slightly reduced EAE-induced BBB disruption, with the improvement becoming statistically significant only in the later stages. Thus, Prdx6 had a beneficial effect on BBB permeability. Fingolimod monotherapy was also effective in the early stage of the disease, and at a later stage, after discontinuation of fingolimod, BBB permeability again increased. The combination of fingolimod+Prdx6 significantly reduced permeability in both the early and late stages.

#### 3.5.2 TJP Levels in the Brain and Spinal Cord

**Fig. (6):**
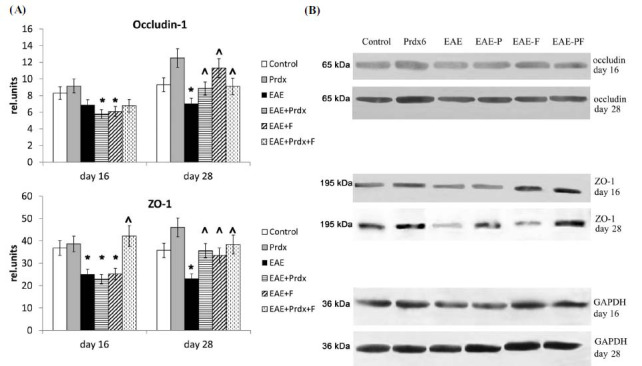
BBB conditions were assessed by occludin-1 and ZO-1 content in the brain (by Western blotting). Experimental groups: healthy mice, Prdx6, EAE, EAE+Prdx6, EAE + fingolimod, and EAE+Prdx6+fingolimod. (**A**) In the histograms, the blot densitometry data were normalized to the corresponding GAPDH loading control and are presented as the means ± SEMs for 3 mice per group. (**B**) The blot images are from a single representative animal per group. Measurements were obtained at different times after immunization (day 0). *Significant difference from the control group, *p* < 0.05. # Significant difference from the EAE group, *p* < 0.05.

**Fig. (7):**
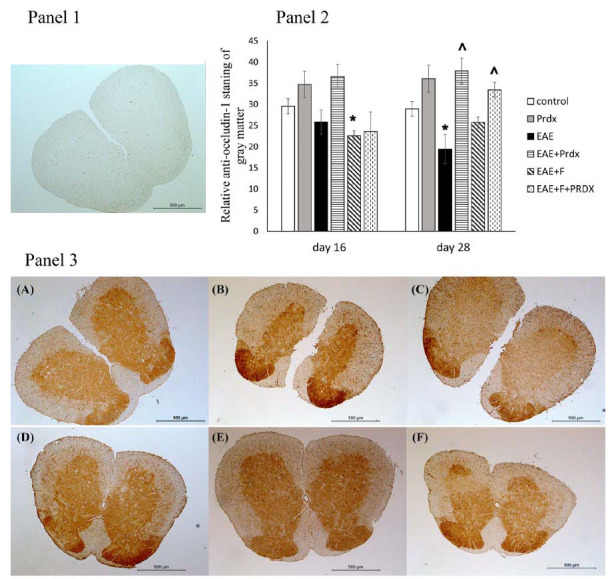
The BBB condition was assessed by anti-occludin-1 staining of spinal cord sections. Experimental groups: (**A**) control (healthy mice), (**B**) Prdx6, (**C**) EAE, (**D**) EAE+Prdx6, (**E**) EAE + fingolimod, and (**F**) EAE+Prdx6+fingolimod. **Panel 1**: The microphotograph shows a negative control section, which was stained in the same way as experimental sections but without the primary antibody. **Panel 2**: The histogram data show the mean pixel intensities of the gray matter area minus the mean pixel intensities of the white matter area in the same section (using ImageJ). For each animal, the values were counted and averaged in three sections (technical repeats). The data presented are the means ± SEMs for 3 individual mice per group. *Significant difference from the control group, *p* < 0.05. ^ Significant difference from the EAE group, *p* < 0.05. **Panel 3**: Each microphotograph shows an example from a single representative animal per group on day 28 of the experiment.

We also measured the levels of the Tight Junction Proteins (TJPs) occludin-1 and ZO-1 in the brain *via* Western blotting (Fig. **6**) and occludin-1 in the spinal cord *via* immunohistochemistry (Fig. **7**).

The Western blotting results revealed a significant decrease in the levels of occludin-1 and ZO-1 in the brain tissue of mice with EAE at the early and late stages of the disease. In the spinal cord, similar changes in occludin-1 content were observed only at the later stage of the disease. The administration of fingolimod to EAE mice tended to accelerate the decrease in occludin-1 levels in the spinal cord, although the discontinuation of fingolimod resulted in the restoration of normal TJP levels.

Prdx6 monotherapy improved ZO-1 levels in the brain, as well as occludin-1 levels in the spinal cord, at the later stage of EAE. The use of Prdx6 in combination with fingolimod reduced the decrease in occludin-1 and ZO-1 levels, restoring them to normal levels and improving the state of the BBB, and this combination produced the best outcome among the studied groups.

### 3.6 Expression of Genes Involved in the Inflammatory Response and Oxidative Stress in Brain Tissue

**Fig. (8):**
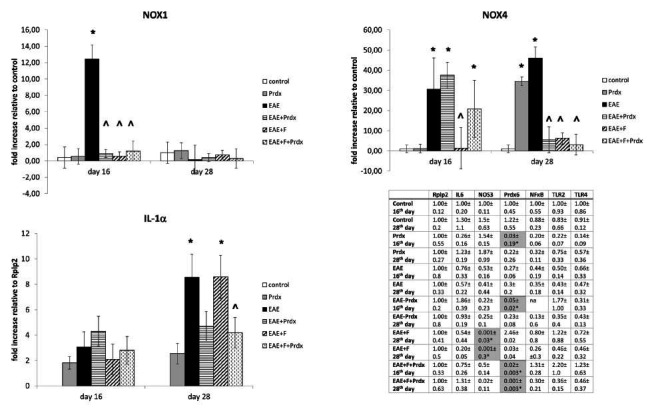
Expression of inflammatory response- and oxidative stress-related genes in brain tissue by qRT‒PCR. Experimental groups: healthy mice, Prdx6, EAE, EAE+Prdx6, EAE + fingolimod, and EAE+Prdx6+fingolimod. Measurements were obtained at different times after immunization (day 0). The values averaged from three independent measurements were normalized to the expression of the *Rplp2* housekeeping gene and then to the corresponding gene expression in healthy controls or *Rplp2* expression only (for IL-1alpha, because of its undeterminable level in both control groups) and expressed as a fold change. The plots show the most prominent (+)-changes observed for NOX1, NOX4, and IL1α genes, whereas the table shows the measured expression for genes with low-grade or no changes or (-)-changes (gray). *Significantly different from the control group, *p* < 0.05; ^ significantly different from the EAE group.

Mechanisms of the BBB breakage, as well as of the protective effect of Prdx6 and fingolimod, were assessed by measurements of the expression of genes associated with the inflammatory response and oxidative stress in the brain tissue of the mice *via* quantitative real-time RT‒PCR (Fig. **8**).

Among the genes related to inflammation, only a gene encoding interleukin-1 alpha exhibited a noticeable variation in expression in the brain tissue of the EAE mice compared with those of healthy controls. There was no significant (in our case, more than 5-fold) increase in the expression of such genes as interleukin-6, TNF-alpha, NOS3, or the Toll-like receptors *TLR2* and *TLR4* genes. In fact, many of the markers showed a tendency toward inhibition. However, it should be noted that the level of IL-1-alpha in the EAE+fingolimod group increased sharply at the later stage, also demonstrating the proinflammatory “fingolimod withdrawal effect,” while coadministration of Prdx6 and fingolimod reduced this phenomenon.

The levels of the NADPH oxidases *NOX1* and *NOX4*, which are highly expressed in neural tissues, unlike *NOX2*, were greatly increased (10- to 100-fold) in EAE mice compared with healthy controls. *NOX1*, an inducible oxidase, exhibited a dramatic increase in expression in the early stage of EAE and later decreased to control levels. The expression of *NOX4,* which may be called both a constitutive and inducible oxidase, increased tenfold in the early phase and about one hundred-fold in the later phase. The administration of fingolimod or Prdx6, separately, as well as in combination, completely blocked EAE-induced *NOX1* expression. *NOX4* expression, which is elevated in EAE, was reduced by Prdx6 at the later stage but not at the early stage, whereas fingolimod blocked *NOX4* expression at both the early and later stages. Coadministration of Prdx6 and fingolimod also blocked *NOX4* expression at both stages.

Thus, the increased expression of NADPH oxidases in EAE, which may be one of the possible mechanisms of BBB destruction, was diminished by Prdx6, making it possible to neutralize BBB disruption, exert a beneficial health effect, and reduce the fingolimod withdrawal effect.

### 3.7 Lymphocyte Infiltration into the Spinal Cord

**Fig. (9):**
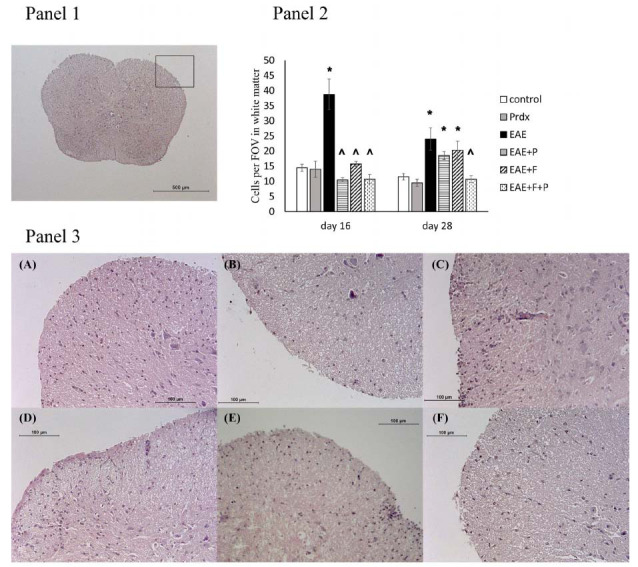
Lymphocyte infiltration into the spinal cord was assessed by hematoxylin-eosin staining. Experimental groups: (**A**) control (healthy mice), (**B**) Prdx6, (**C**) EAE, (**D**) EAE+Prdx6, (**E**) EAE + fingolimod, and (**F**) EAE+Prdx6+fingolimod. **Panel 1**: The photograph shows a lower-magnification image (scale bar 500 μm), where the approximate position for the bottom higher magnification (scale bar 100 μm) region is indicated. **Panel 2**: For each animal, the cells in three Fields Of View (FOVs) in a region of white matter were counted and averaged. The histogram data show the means ± SEMs for 3 individual mice per group on days 16 and 28 of the experiment. *Significant difference from the control group, *p* < 0.05. ^ Significant difference from the EAE group, *p* < 0.05. **Panel 3**: Each microphotograph shows a spinal cord section from a single representative animal per group (**A-F**) on day 28 of the experiment.

To assess the structural integrity of the BBB and the ability of immune cells to penetrate nervous tissue, a histological assessment of sections of the spinal cord from mice was performed using hematoxylin-eosin staining (Fig. **9**).

Our results demonstrated that EAE induced significant lymphocyte infiltration into the white matter of the spinal cord compared with healthy controls. At the later stage of the disease, infiltration decreased to some extent but remained elevated. The administration of Prdx6 or fingolimod, as well as their combination, effectively reduced the early lymphocyte counts observed in the white matter of EAE mice, which was consistent with their beneficial effect on BBB status. However, in the fingolimod-treated group, after the discontinuation of fingolimod, lymphocyte infiltration increased again, which was consistent with the increase in BBB permeability. Administration of fingolimod in combination with Prdx6 prevented the effect of fingolimod withdrawal on the penetration of lymphocytes into the nervous tissue.

## 4 DISCUSSION

### 4.1 Prdx6 and Fingolimod Improved BBB Condition in EAE

Disruption of the blood-brain barrier in multiple sclerosis and EAE is likely one of the main causes of the onset and progression of these autoimmune diseases since it involves the infiltration of lymphocytes into the central nervous system and their attack on the myelin sheaths, which mediate the most characteristic symptoms of multiple sclerosis. Restoring the damaged BBB should weaken if not completely halt, the inflammatory process in the nervous system. Aligning with our previous results, this present study demonstrates [47] that the antioxidant enzyme peroxiredoxin-6, which, along with peroxidase activity, also has phospholipase activity [41], exerts a very beneficial effect on the state of the BBB. Nearly all the mice with EAE treated with a total of 4 injections of this protein experienced complete symptom relief. This effect correlated with an improvement in the permeability of the BBB, so it can be concluded that the beneficial effect on the health status of mice with EAE may, at least in part, have been due to its effects on the blood-brain barrier. The mechanism of the effect on the BBB may be associated with the inhibitory effect of Prdx6 on the expression of the NADPH oxidases NOX1 (inducible oxidase) and NOX4 (both constitutive and inducible oxidase), which we identified. These oxidases are dramatically increased in the brains of mice with EAE, and the reactive oxygen species they produce are known to disrupt the integrity of the BBB directly [54]. The activation of NOX in neurodegenerative diseases has been shown previously [55] and was confirmed by our data, showing increases in expression by 10-fold (for *NOX1*) and 100-fold (for *NOX4*) in EAE. The nearly complete blockade of this increased expression by peroxiredoxin-6, which correlated with improvements in BBB status measured using Evans blue dye, TJP levels, and lymphocyte infiltration, may reflect a mechanism for BBB improvement.

Our data did not allow us to directly identify a specific cause of such sharp decreases in *NOX* expression in response to peroxiredoxin-6, but a decrease in *NOX* expression should reduce the amount of reactive oxygen species production. According to the literature, the relationship between Prdx6 and NOX is quite complex. Previous studies found that Prdx6 participates in the activation of NOX2 in human neutrophils, alveolar macrophages, endothelial and other cell types [56-59]. Prdx6 catalyzes the removal of H_2_O_2_ and other hydroperoxides [60]. Additionally, NOX1 activation depends on interactions with cytosolic regulatory cofactors, including Rac1 [61-63], Nox organizer 1 (Noxo1), a p47phox adapter protein homolog, and Nox activator 1 (Noxa1), a p67phox activator protein homolog [64-66]. There is evidence that the phospholipase (PLA2), but not peroxidase activity of Prdx6 is necessary for NOX2 activation in macrophages and the lung epithelium and that NOX2 activation does not occur in Prdx6 knockout animals [56-58]. It has also been shown that Prdx6 in cells directly binds to NOX activator 1 (Noxa1) and increases the production of superoxides by NOX1 oxidase through its PLA2 activity, while both peroxidase- and lipase-deficient mutant forms of Prdx6 (Prdx6 C47S and S32A, respectively) failed to bind to or stabilize NOX1 components or support NOX1-mediated superoxide generation [67]. Moreover, Prdx6 expression is increased in the spinal cord of mice with EAE, and compared with normal mice, transgenic mice with increased expression of Prdx6 have a lower severity of symptoms and rate of demyelination [39]. Thus, the three-level regulation of the expression of NADPH oxidases by Prdx6 is as follows: firstly, Prdx6 is necessary for the activation of NOX proteins; secondly, it suppresses the induction of *NOX* gene expression; and thirdly, it is an antioxidant enzyme. It should be noted that the above results describe the roles of the *endogenous* Prdx6 in interaction with NOXes. Mechanisms of the observed effect of exogenous Prdx6 are of particular interest.

Our previous results showed that in the first hour after injection of Prx6, around 80% of the initial amount of the protein is present in the blood. The content of exogenous Prx6 decreases with time by about 3 times in 6 hours [68]. Similar results were obtained for Prdx1 [69]. Therefore, the exposure of Prdx6 in circulation is rather high. Along with that, we previously showed that exogenous Prdx6 is able to penetrate cells, localizing in cytoplasm. Mechanisms of this effect are likely to be associated with phospholipase A2 activity of Prdx6 because mutated Prdx6, lacking PLA2 activity, was unable to penetrate [70]. We have no data on direct contacts between exogenous Prdx6 and the activated membrane NOX complexes, although such interaction cannot be excluded. However, accounting for the above data on Prdx6 penetration into cells, we now suggest that a short-term increase of the exogenous Prdx6 in the cytoplasm may suppress the production of endogenous Prdx6 *via* an unknown mechanism (for example, as a feedback loop). We observed this effect in the present study by the measurement of the *Prdx6* mRNA). Suppression of endogenous Prdx6, if it is long-lasting, may prevent new NOX1 complexes activation in an autoimmune environment because endogenous Prdx6 binding to NOX activator 1 (Noxa1) and assembling into new NOX complex are necessary for NOX activation [67]. It should be noted that his mechanism is hypothetical, and new data is required to confirm it. Therefore, exogenous Prdx6 may act as an antioxidant in the extracellular environment and simultaneously inhibit the expression of endogenous Prdx6 in cells, contributing to the blockade of NOX1 through a decrease in the binding of endogenous Prdx6 to Noxa1.

We also examined the effects of fingolimod, an agonist and functional antagonist of S1P receptors, which is currently among the most advanced clinically used drugs for the treatment of multiple sclerosis, on preventing the release of lymphocytes from peripheral lymphoid organs [17]. As noted in the Introduction, we believe that the pleiotropic effect of fingolimod through S1P receptors, along with the anti-lymphocyte effect, may contribute to the deterioration of the BBB [24, 25], and we hypothesized that the use of Prdx6 in combination with fingolimod may reduce the negative side effects. In this study, we were unable to detect any decrease in the barrier function of the BBB caused by fingolimod, probably because of the short period of our study. Fingolimod, which is a clinically effective drug, significantly inhibited lymphocyte infiltration into the CNS, reduced the proinflammatory cytokine response, and had a marked anti-lymphocyte effect. Administration of fingolimod in the setting of EAE did not exacerbate the EAE-induced drop in TJP levels beyond that observed with untreated EAE. Furthermore, the integrity of the BBB, as determined by the Evans blue dye method, was significantly improved by fingolimod, probably due to its anti-inflammatory and anti-lymphocyte effects, which likely reduced the “oxidative burden” on the BBB.

### 4.2 Fingolimod Withdrawal Effect

We discovered that discontinuation of fingolimod was associated with a significant “withdrawal effect,” which was manifested as increased production of proinflammatory cytokines above the levels observed in untreated animals with EAE, increased BBB permeability, increased lymphocyte infiltration into the spinal cord, and deterioration of the general health status, even during the relatively short period of our experiment (1 month). We suggest that the discontinuation of fingolimod likely resulted in a massive release of stress, activated lymphocytes (as seen by their NF-κB status) from the depots, and an increase in symptoms and various EAE-related indicators. This effect was accompanied by a decrease in the activation of the proapoptotic caspase-3 cascade and the antiproliferative and sometimes proapoptotic p53 cascade (*i.e*., lymphocytes restored the ability to survive and proliferate). As a speculation, we suppose that in the autoimmune disease, two counteracting processes take place, *i.e*. the inflammatory damage of BBB and its reparation by defense systems. According to our data, the fingolimod withdrawal obviously led to the worsening of the autoimmune response *via* the release of the previously locked lymphocytes. However, this withdrawal took place nearly the middle of the experiment period, when reparative systems had had time to increase TJP expression in order to repair BBB after its initial breakage by the pertussis toxin during EAE induction. Therefore, during fingolimod withdrawal, we saw increased TJPs along with a new autoimmune attack on BBB, more so that BBB is not fully recovered.

### 4.3 Prdx6 and the Fingolimod Withdrawal Effect

Prdx6, which alone induced activation of lymphocytes and the pro-inflammatory cytokine response, in combination with fingolimod, produced an unexpected and paradoxical effect, counteracting the pro-inflammatory “withdrawal effect” of fingolimod in many aspects. With respect to molecular mechanisms, we suggest that this effect, in addition to the BBB repair, may be associated with Prdx6-induced activation of the proapoptotic (caspase-3) and anti-proliferative (p53) cascades in lymphocytes, resulting in a decline in the lymphocyte count in the blood upon fingolimod withdrawal. Additionally, lymphocytes mostly remained in the spleen depot after discontinuation of fingolimod (compared with those in the EAE+fingolimod group). These results can also be correlated with a downregulation of anti-inflammatory cytokine IL-1-alpha expression in brain tissue in response to Prdx6 during the “withdrawal effect” of fingolimod. It should be noted that the administration of Prdx6 was started and ended before the first administration of fingolimod. This diminishing of the withdrawal effect resulted in an improvement in the general health status of the animals; none of the animals in the EAE+fingolimod+Prdx6 group had visible symptoms of the disease by the end of the experiment.

Current data from clinical studies on drug withdrawal effects during multiple sclerosis have shown that discontinuation of treatment is very likely to lead to disease relapse [71, 72]. As another example, in a cohort study of patients with relapsing-remitting MS, 10% of patients had severe reactivation within 6 months of stopping fingolimod [73]. Treatment of the rebound events after fingolimod withdrawal and the appropriate timing for the next disease management therapy are still inconsistent. Some have advocated pulse steroids, plasma exchanges, or immunoglobulins for the control of fingolimod rebound [74]. The exact mechanism of the rapid lymphocyte re-entry into the CNS remains unknown. Moreover, It should be noted that the fingolimod withdrawal effect was also reported not only in MS but in neuromyelitis optica as well [75]. Therefore, a drug that prevents negative consequences in the case of withdrawal of fingolimod would be very useful.

## 5 STUDY LIMITATIONS

It should be noted that we used EAE as a condition that models MS, and the mechanisms of EAE may differ in some aspects from ones of MS, and a withdrawal effect in MS should be a subject of further investigation.

## CONCLUSION

Our results demonstrated that fingolimod and Prdx6 produced beneficial effects in mice with experimental autoimmune encephalomyelitis, a model of multiple sclerosis. Either fingolimod or Prdx6 as monotherapy improved the overall health status of mice with EAE and improved their BBB function. In the case of fingolimod, locking lymphocytes in lymph nodes, the effect on the BBB was probably related to the decrease of oxidative burn on the BBB, facilitating its reparation by defense systems. In the case of Prdx6, the effect on BBB was probably related to the modulation of expression of NADPH oxidases in brain tissues.

Furthermore, our data indicated that discontinuation of fingolimod produced a “withdrawal effect,” manifesting in worsened symptoms, increased autoimmune inflammatory response, and impairment of the BBB condition. The exact mechanisms of this effect remain to be elucidated, though our study indicates that they may be associated with immune cell activation when they leave lymph node depots where the anti-lymphocyte drug locks them. Peroxiredoxin 6, administrated before fingolimod withdrawal, improved BBB condition and health status of animals with EAE and ameliorated the fingolimod withdrawal effect, probably *via* activation of anti-proliferative and pro-apoptotic pathways in spleen cells, leading to full recovery from EAE.

## DISCLOSURE

The present study is a continuation of the referenced work [28]. Several methods are common, and their descriptions are inevitably similar to the previously published article “Protective effect of exogenous peroxiredoxin 6 and thymic peptide thymulin on BBB conditions in an experimental model of multiple sclerosis”, in Archives of Biochemistry and Biophysics Volume 746, 15 September 2023, 109729.

## Data Availability

The data and supportive information are available within the article.

## LIST OF ABBREVIATIONS

BBB: Blood-Brain Barrier
EAE: Experimental Autoimmune Encephalomyelitis
MS: Multiple Sclerosis
PLP: Proteolipid Protein
S1P: Sphingosine-1-phosphate
TJP: Tight Junction Protein
ZO-1: Zonula Occludens
